# Decline in Motor Function during the COVID-19 Pandemic Restrictions and Its Recovery in a Child with Cerebral Palsy: A Case Report

**DOI:** 10.3390/children8060511

**Published:** 2021-06-17

**Authors:** Daiki Asano, Naoko Kikuchi, Toru Yamakawa, Shu Morioka

**Affiliations:** 1 Department of Rehabilitation, Japan Baptist Hospital, Kyoto 606-8273, Japan; 2 Department of Pediatrics, Japan Baptist Hospital, Kyoto 606-8273, Japan; naoko.kikuchi@jbh.or.jp (N.K.); toru.yamakawa@jbh.or.jp (T.Y.); 3 Neurorehabilitation Research Center, Kio University, Nara 635-0832, Japan

**Keywords:** cerebral palsy, home confinement, COVID-19, motor function, recovery

## Abstract

Children with cerebral palsy (CP) experience various restrictions owing to their underdeveloped mobility. Home confinement due to the coronavirus disease 2019 pandemic may further increase these restrictions. We report the case of a 7-year-old boy with CP (Gross Motor Function Classification System level IV) whose motor function declined during the period when physical therapy was discontinued due to lockdown, approximately four months. At the end of the home confinement, the patient’s ability to maintain a sitting posture and weight-bearing capacity of the lower extremities decreased. His Gross Motor Function Measure total score also decreased from 34.5% to 31.9%. After resuming physical therapy, the patient recovered the function status seen before the discontinuation of physical therapy, but this took almost twice as long as the confinement period. We reaffirm that frequent physical therapy is crucial for maintaining motor function in non-ambulatory children with CP. As a countermeasure for the future, urgent efforts are needed for the development of telerehabilitation.

## 1. Introduction

The spread of novel coronavirus disease 2019 (COVID-19) has forced many children into home confinement. In Japan, all schools were closed from February to May 2020 to prevent COVID-19 outbreaks. Leaving home was forbidden except for non-deferrable work or for health reasons that required treatment for illness; all non-essential services and manufacturing were stopped; all sports and artistic events were cancelled. Notably, after-school educational and recreational activities for children were suspended, and the educational and therapeutic services directed to children with special needs of all ages were closed. The impact of home confinement due to the COVID-19 pandemic on children’s behavior and mental health has been reported in several studies not only in children with typical development but also in children with developmental disabilities [1,2,3]. In addition, a recent study reported that imposed movement restrictions had a negative effect on children’s motor competence development [4]. However, there are no reports on the impact of home confinement in children with movement disorders. Previous studies have pointed out that the motor function of children with cerebral palsy (CP), a motor impairment caused by perinatal brain injury, is particularly sensitive to changes in environmental factors [5,6,7]. It has also been reported that there is a significant relationship between motor function and the intensity of participation in recreational and leisure activities in children with CP [8]. Based on the above evidence, living in home confinement may adversely affect the motor function of children with CP because they are not allowed to participate in outdoor activities or visit hospitals for physical therapy.

We reported the case of a child with CP whose motor function decreased due to home confinement related to the COVID-19 pandemic, along with a description of the subsequent recovery process. Written informed consent was obtained from the patient’s parents for the publication of this case report.

## 2. Case Presentation

The patient was a 7-year-old boy with spastic quadriplegic CP. He was born by spontaneous vaginal delivery at 33 weeks of gestation, had a birth weight of 2060 g, and had Apgar scores of 8 and 8 at 1 and 5 min, respectively. After birth, he was transferred to the neonatal intensive care unit (NICU) and managed in an incubator for 2 weeks. Brain magnetic resonance imaging before discharge revealed bilateral cystic periventricular leukomalacia. The patient was discharged from the NICU at 38 weeks of gestation and started on physical therapy for 90 min once a week at the outpatient clinic of Japan Baptist Hospital at 2 months of chronological age.

**Figure 1:**
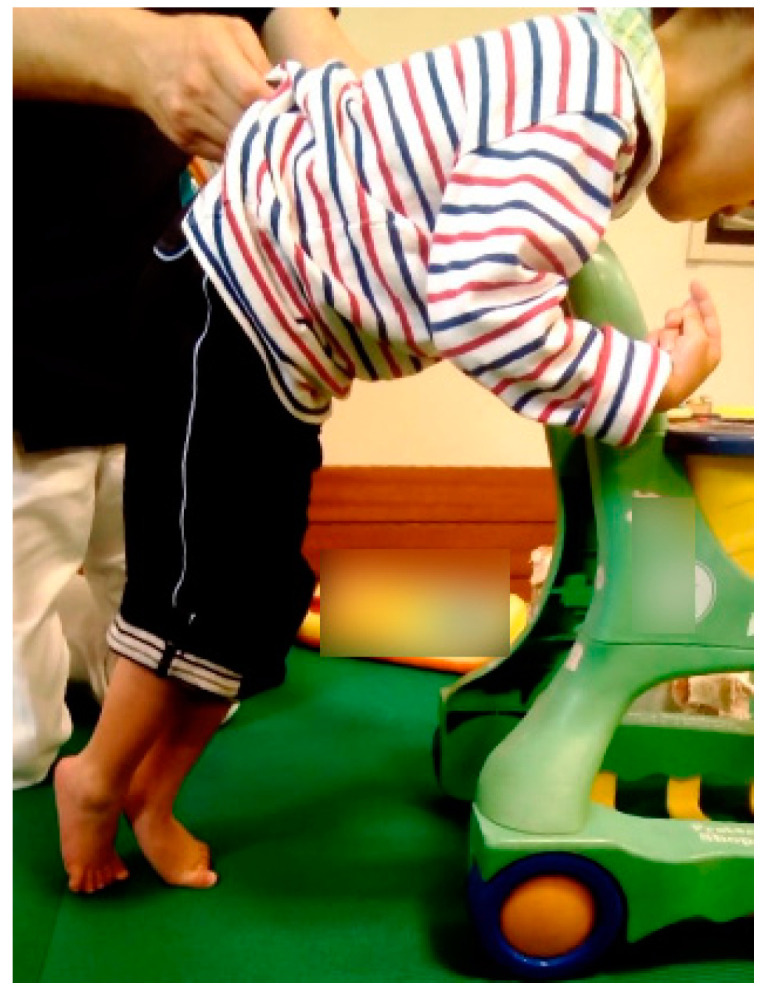
Standing posture of the patient at age 4 years.

At 2 years and 6 months of age, he acquired the ability to roll over, and his Gross Motor Function Classification System (GMFCS) level was level IV. He had moderate spasticity of the modified Ashworth scale level 2 in both the lower extremities and right upper extremity, but the left upper extremity showed relatively mild paralysis, with his left hand being the dominant one. He intentionally grasped and released objects with only his left hand. When he tried to stand up, his lower limbs and equinus foot showed a scissors position (Figure 1). There were no problems with the range of motion. The patient also had no swallowing or respiratory problems. However, he had impairment of the oculomotor system, such as strabismus, abnormal fixation, and following movements disorder. At 4 years and 9 months of age, he had acquired the ability to maintain a sitting position on a bench for several minutes. Goal attainment scaling (GAS) [9,10] was used as an outcome measure for subsequent walking practice using parallel bars (Table 1). Walking practice with a walker was also initiated at the same time.

At 6 years and 3 months of age, assessment with the Vineland Adaptive Behavioral Scales, second edition (Vineland-II) showed a standard score of 30 for communication (receptive language skills, age equivalent = 14 months; expressive language skills, age equivalent = 21 months), 27 for daily living skills (personal skills, age equivalent = 18 months; community skills, age equivalent = 22 months), 59 for socialisation (interpersonal relationship, age equivalent = 13 months; play and leisure time, age equivalent = 18 months; coping skills, age equivalent = 29 months), and 20 for motor skills (gross motor skills, age equivalent = 7 months; fine motor skills, age equivalent = 16 months). The Vineland-II is the most commonly used instrument for quantifying impairments in adaptive behaviors necessary for socialization, communication, and daily functioning [11]. In terms of the patient’s motor function, head movement was well controlled; however, maintaining a sitting posture on a chair or on the floor during hand use and the reaching movement was at a level that required monitoring. Hand manipulation was mainly done with the left hand, and power grasping and pinching with three fingers were possible.

At the age of 6 years and 4 months, he was enrolled in a special-needs school that he was scheduled to attend, but the school was closed for 2 months due to the COVID-19 pandemic. In addition, weekly physical therapy was suspended for 4 months on account of the lockdown. According to his mother, during home confinement, the patient continued to perform daily activities in a manner like that before the lockdown, except that he did not go outdoors. When physical therapy was subsequently resumed, the patient’s ability to maintain a sitting posture and weight-bearing capacity of the lower extremities were found to have decreased, and the Gross Motor Function Measure (GMFM-88) total score decreased from 34.5% to 31.9% (Table 2). This decline in scores was more pronounced in the standing domain (dimension D) than in the other domains and was higher than the minimum clinically important difference (MCID) value of 5.2%, recently reported by Storm et al. [12]. Specifically, the scores of items 53 to 55, related to the ability to maintain a standing position, decreased. The GAS score decreased from 1 to −2. There was no change in the scale scores of any of the Pediatric Evaluation of Disability Inventory (PEDI) domains during the lockdown (Table 2). His standing posture and assisted walking opportunities were lost during the lockdown, and his ability to maintain a standing position was thought to have decreased due to the reduced amount of experience.

**Figure 2:**
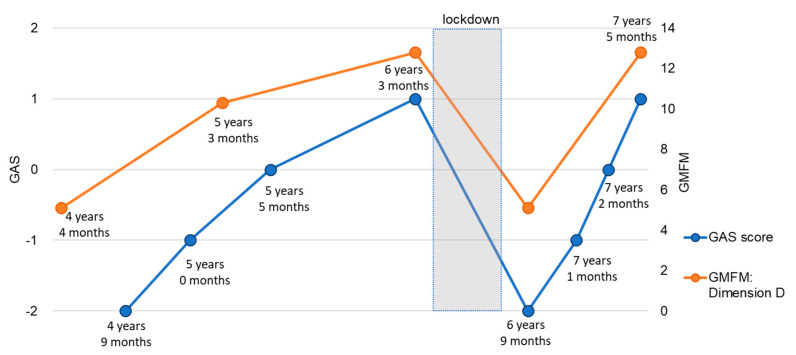
Changes in GAS score and GMFM dimension D.

After resuming physical therapy once a week, with dynamic sitting and standing posture control and gait training, the GAS score recovered to −1 after 4 months involving 11 training sessions, recovered to a score of 0 after another month involving five sessions, and changed to a score of 1 after 3 months involving six sessions (Figure 2). The GMFM-88 score also recovered to the same level as that before home confinement (Table 2, Figure 1).

## 3. Discussion

In this report, we presented the case of a child with CP whose motor function regressed due to home confinement related to the COVID-19 pandemic. A home confinement period of almost 4 months caused a decline in his ability to maintain a standing or sitting posture. After the resumption of physical therapy, it took 8 months of weekly intervention for the patient’s motor function to return to its previous state. It is interesting to note that the recovery of motor function took almost twice as long as the time taken for its decline.

In patients with brain injury, activity-dependent plasticity occurs in the motor cortex, and thus, intensive and repetitive task-specific exercises can be used to improve motor recovery [13,14,15]. Moreover, many children with CP experience restrictions in daily activities and social participation, and these problems are mainly attributed to limited gross motor function, low levels of education, and young age [16]. In this case, in addition to the restrictions in daily activities and participation, home confinement due to the COVID-19 pandemic further deprived the patient of opportunities for therapeutic exercise, which may have contributed to the decline in motor function. In the future, it will be essential to provide training programs that can be conducted at home and to set up telerehabilitation facilities in preparation for another lockdown. Recent studies have shown the possibility of setting up and delivering home-based telerehabilitation services and their effectiveness [17,18,19]. Decreased physical activity also causes musculoskeletal problems, such as joint contractures, in children with CP. This patient corresponds to stage 1 in which abnormal postures are dynamic without contractures, according to the recently reported classification for lower limbs musculoskeletal pathology in children with CP [20]. It will be necessary to maintain the amount of physical activity to prevent the occurrence of secondary contractures.

In this study, the dimensional scores of the GMFM-88 gave us detailed information on the motor function decline in this patient. In children with severe cerebral palsy classified as GMFCS level IV or V, the GMFM-88, which has more items for evaluation in the supine and sitting positions, may be more appropriate.

Regarding the frequency of intervention, it has been found that more frequent interventions are necessary to improve gross motor function [21]. In the present study, although an intervention spanning approximately 90 min once a week helped the patient regain his motor functions, increasing the intensity might have helped him recover more rapidly. Home-based telerehabilitation can be an effective tool in terms of improving intervention frequency because it provides an opportunity to increase the frequency of skills training.

As a limitation, we did not evaluate mental health or behavioral problems in this case. Previous research has shown that the restriction of various daily and outdoor activities can lead to mental health problems, such as depressive symptoms, in children with CP [22]. Particularly, decline of activities and enjoyment in their daily life was identified as a contributing factor to greater severity of depressive symptoms [23]. Therefore, it is necessary to pay attention to changes in mental health and behavior and take appropriate action as soon as signs are detected in such children. Another fundamental limitation is that we cannot determine the extent to which the physical therapy intervention contributed to the recovery of this patient. However, because this patient had few opportunities to stand and walk in his daily life, it is highly possible that training that was focused on standing and walking contributed to the recovery of the patient’s decreased motor functions. In addition, the patient’s mother reported no significant change in his activities of daily living before and after the resumption of physical therapy, suggesting the need for intervention to maintain motor function.

In summary, the motor function of children with CP is vulnerable to the negative effects of home confinement due to the COVID-19 pandemic, and regular remote or direct intervention by professionals is needed to prevent functional decline in the future. Home-based telerehabilitation is an important option for this purpose.

## 4. Conclusions

This report suggests that non-ambulatory children with CP are more susceptible to lockdown-induced deprivation of activity opportunities. In addition, the time required for the recovery of motor function is approximately two-times longer than the time required for motor function decline. Hence, in the future, the establishment of telerehabilitation will be crucial as a countermeasure.

## Figures and Tables

**Table 1:** Goal setting in this case using the goal attainment scaling (GAS).

Level of Attainment	Goal (Amount of Assistance Needed during Walking)
Much less than expected −2 (Pre-intervention status)	During walking, with both hands grasping the support (right hand is assisted), the patient requires assistance in adjusting the amount of lower limb load, holding the pelvis to maintain balance, moving the left hand, and correcting the stepping foot position.
Somewhat less than expected −1	During walking, the lower limbs do not collapse, and assistance in adjusting the amount of load is no longer necessary.
Expected level of outcome 0	During walking, the patient no longer needs assistance in moving the left-hand position.
Somewhat more than expected +1	During walking, the patient can correct the position of the stepping foot by himself.
Much more than expected +2	During walking, the patient requires assistance only with his right hand, which is his non-dominant hand.

**Table 2:** Developmental course and changes in motor function of the patient.

Assessment	1st	2nd	3rd	4th	5th	6th	7th	8th
Age	1 Year 9 Months	2 Years 3 Months	3 Years 3 Months	4 Years 4 Months	5 Years 3 Months	6 Years 3 Months	6 Years 9 Months	7 Years 5 Months
GMFCS level	-	V	IV	IV	IV	IV	IV	IV
MACS level	-	-	-	IV	IV	III	III	III
CFCS level	-	-	-	IV	IV	IV	IV	IV
GMFM-66	25.3	31.2	35.7	36.4	39.5	40.9	39.0	40.9
GMFM-88 total	18.7	21.5	26.3	27.4	32.0	34.5	31.9	34.5
A: Lying, rolling	78.4	78.4	80.4	82.4	86.3	90.2	90.2	90.2
B: Sitting	15.0	26.7	40.0	43.3	53.3	56.7	55.0	56.7
C: Crawling, kneeling	0.0	2.4	4.8	4.8	4.8	7.1	4.8	7.1
D: Standing	0.0	0.0	5.1	5.1	10.3	12.8	5.1	12.8
E: Walking, running, jumping	0.0	0.0	1.4	1.4	5.6	5.6	4.2	5.6
PEDI (scaled score)								
Self-Care	NA	NA	NA	NA	NA	39.6	39.6	39.6
Mobility	NA	NA	NA	NA	NA	30.6	30.6	30.6
Social function	NA	NA	NA	NA	NA	34.0	34.0	34.0

GMFCS, Gross Motor Function Classification System; MACS, Manual Ability Classification System; CFCS, Communication Function Classification System; GMFM, Gross Motor Function Measure; PEDI, Pediatric Evaluation of Disability Inventory; NA, not available.

## Data Availability

All relevant data are within the manuscript.
